# Post-translational modifications and bronchopulmonary dysplasia

**DOI:** 10.3389/fped.2024.1426030

**Published:** 2025-01-03

**Authors:** Kun Yang, Ting He, Xue Sun, Wenbin Dong

**Affiliations:** Department of Neonatology, Children’s Medical Center, The Affiliated Hospital of Southwest Medical University, Luzhou, China

**Keywords:** post-translational modification, bronchopulmonary dysplasia, hyperoxia, lung injury, substrate protein

## Abstract

Bronchopulmonary dysplasia is a prevalent respiratory disorder posing a significant threat to the quality of life in premature infants. Its pathogenesis is intricate, and therapeutic options are limited. Besides genetic coding, protein post-translational modification plays a pivotal role in regulating cellular function, contributing complexity and diversity to substrate proteins and influencing various cellular processes. Substantial evidence indicates that post-translational modifications of several substrate proteins are intricately related to the molecular mechanisms underlying bronchopulmonary dysplasia. These modifications facilitate the progression of bronchopulmonary dysplasia through a cascade of signal transduction events. This review outlines the relationships between substrate protein phosphorylation, acetylation, ubiquitination, SUMOylation, methylation, glycosylation, glycation, S-glutathionylation, S-nitrosylation and bronchopulmonary dysplasia. The aim is to provide novel insights into bronchopulmonary dysplasia's pathogenesis and potential therapeutic targets for clinical management.

## Introduction

Bronchopulmonary dysplasia (BPD) represents a chronic lung ailment affecting premature infants, characterized by complex pathogenesis and clinical manifestations associated with elevated morbidity and mortality rates (1). Despite remarkable advancements in perinatal medicine, understanding the pathophysiology of BPD remains a formidable challenge. Therefore, investigating BPD's pathogenesis from multiple perspectives is of paramount importance. Post-translational modifications (PTMs) are distinct chemical alterations occurring at amino acid termini or side chains of proteins subsequent to translation. These modifications bestow proteins with high complexity and diverse biological functions. Over 200 PTMs have been identified, most of which are catalyzed by enzymes, with some being non-enzymatic reactions (2). Table 1 lists the characteristics of several common PTMs. A growing body of evidence indicates that PTMs play a significant role in the development of various diseases associated with prematurity, with BPD being of particular interest (6, 7). This review delves into the potential relationships between well-studied PTMs such as phosphorylation, acetylation, ubiquitination, and methylation, and the development of BPD. Beyond elucidating the fundamental principles of these PTMs, the focus lies in elucidating how these modifications influence specific substrate protein characteristics, thus impacting BPD. Understanding the molecular mechanisms of protein PTMs holds significance as it may unveil new avenues for therapeutic strategies in managing BPD.

**Table 1:** Characteristics of several common PTMs (3–5).

PTMs	Modifying group	Modifying group types	Modification location	Key enzymes	Cell biological processes involved
Phosphorylation	Phosphate group	Small chemical group	Serine, threonine and tyrosine	Kinases and phosphatases	Signal transduction, protein interactions and subcellular localization
Acetylation	Acetyl group	Small chemical group	Lysine	Acetyltransferases and deacetylases	Transcription, protein degradation, stress response and cellular metabolism
Methylation	Methyl group	Small chemical group	Lysine and arginine	Methyltransferases	Cell differentiation, transcription and DNA damage response
Succinylation	Succinyl group	Small chemical group	Lysine	SIRT5	Metabolic homeostasis and epigenetic regulation
Ubiquitination	Ubiquitin	Polypeptide chain	Lysine	E1 activating enzymes, E2 conjugating enzymes, E3 ligating enzymes and DUBs	Cell cycle, proliferation, differentiation, apoptosis and DNA damage repair
Neddylation	NEDD8	Polypeptide chain	Lysine	E1-E2-E3 multi-enzyme system and deneddylases	Protein interactions, signal transduction, cell cycle and autophagy response
SUMOylation	SUMO	Polypeptide chain	Lysine	E1 activating enzyme (SAE1/SAE2), E2 conjugating enzyme (Ubc9), E3 ligation enzymes and SENPs	Cell cycle, DNA damage repair, protein transcription, interactions and inter-nucleoplasmic transport
Glycosylation	Glycans	Complex molecules	Serine or threonine	Glycosyltransferase and glycosidases	Protein secretion, transport, folding and degradation
Glycation	Glucose	Complex molecules	Inconclusive	Non-enzymatic reaction	Reduces protein activity
Lipidation	Lipid groups	Complex molecules	Inconclusive	Lipotransferases	Protein transport, location and interactions
Prenylation	Isoprenoid lipids	Complex molecules	Cysteine	FPPS and GGPPS	Signal transduction
S-Glutathionylation	Glutathione	Complex molecules	Cysteine	GST and Grx	Redox regulation
S-Nitrosylation	Nitrosyl group	Small chemical group	Cysteine	GSNOR and Trx	Redox regulation

PTMs, post-translational modifications; SIRT5, sirtuin 5; DUBs, deubiquitinating enzymes; NEDD8, neural precursor cell-expressed, developmentally downregulated 8; SUMO, small ubiquitin-like modifier; SENPs, SUMO-specific proteases; FPPS, farnesyl pyrophosphate synthase; GGPPS, geranylgeranyl pyrophosphate synthase; GST, glutathione S-transferase; Grx, glutaredoxin; GSNOR, S-nitrosoglutathione reductase; Trx, thioredoxin.

### Phosphorylation

**Figure 1:**
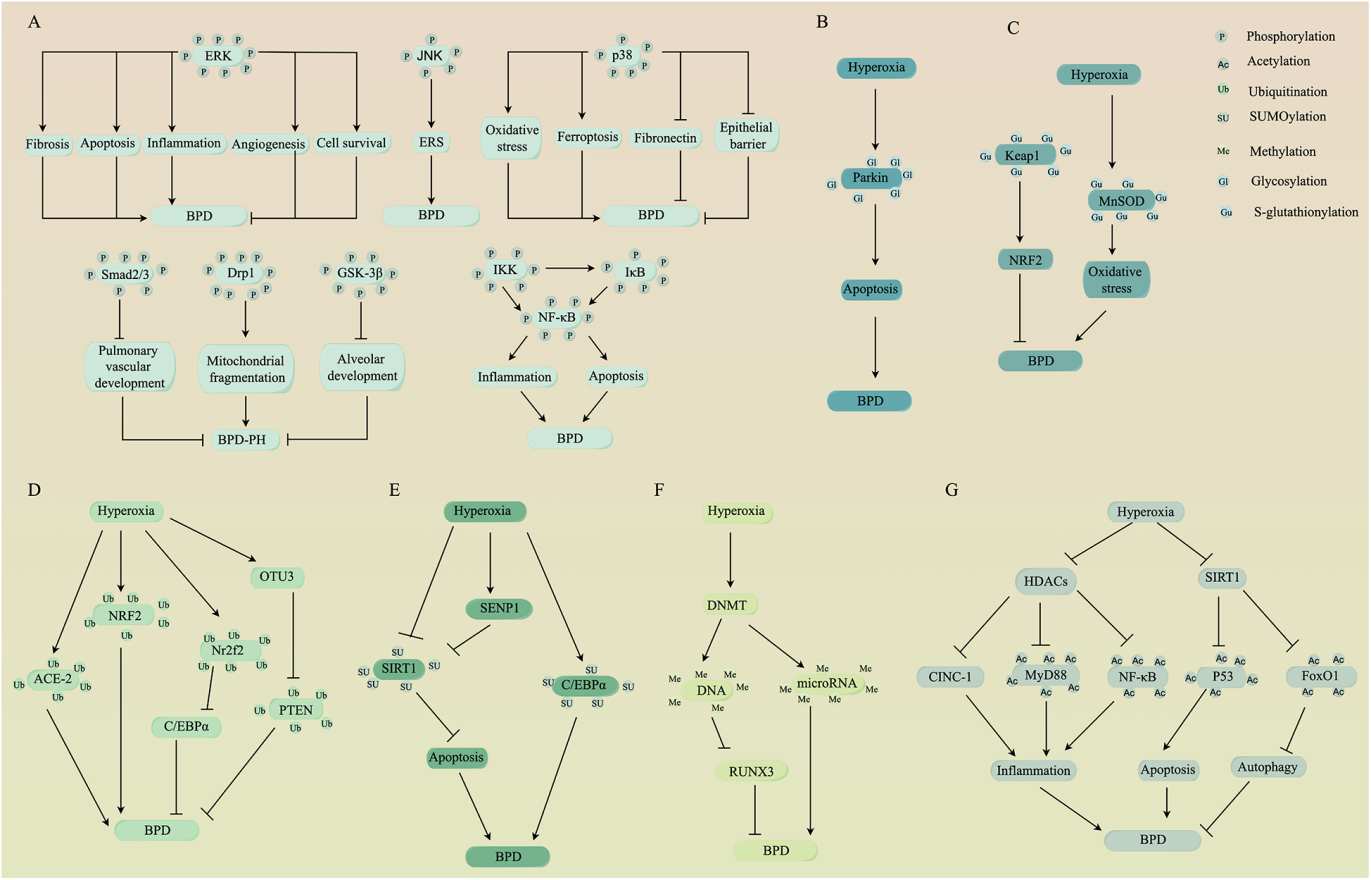
PTMs of several substrate proteins are involved in the pathogenesis of BPD. PTMs including **(A)** phosphorylation, **(B)** glycosylation, **(C)** S-glutathionylation, **(D)** ubiquitination, **(E)** SUMOylation, **(F)** methylation, and **(G)** acetylation alter the activity of substrate proteins, which can positively or negatively affect BPD through a series of signal transduction events. Schematic created by Figdraw (www.figdraw.com). PTMs, post-translational modifications; BPD, bronchopulmonary dysplasia; ERK, extracellular signal-regulated kinase; JNK, c-Jun N-terminal kinase; ERS, endoplasmic reticulum stress; Smad, small mothers against decapentaplegic; Drp1, dynamin-related protein 1; BPD-PH, BPD-pulmonary hypertension; GSK-3β, glycogen synthase kinase-3β; IκB, inhibitor of κB; IKK, inhibitor of κB kinase; NF-κB, nuclear factor-kappa B; Keap1, kelch-like ECH-associated protein 1; NRF2, nuclear factor erythroid 2-related factor 2; MnSOD, manganese superoxide dismutase; ACE-2, angiotensin-converting enzyme-2; Nr2f2, nuclear receptor subfamily 2, group F, member 2; C/EBPα, CCAAT/enhancer-binding protein alpha; OTUs, ovarian tumor proteases; PTEN, phosphatase and tensin homolog; SIRT1, sirtuin 1; SENPs, SUMO-specific proteases; DNMT, DNA methyltransferase; RUNX3, runt-related transcription factor 3; HDACs, histone deacetylases; CINC-1, cytokine-induced neutrophil chemoattractant-1; FoxO1, forkhead box O1.

Phosphorylation, one of the most common PTMs, plays a crucial role in various cellular processes. It influences substrate protein interactions, enzyme activity, subcellular localization, half-life and the turnover of target proteins through kinases (which add phosphate groups via phosphorylation) and phosphatases (which remove phosphate groups via dephosphorylation). Phosphorylation predominantly occurs at serine, threonine, and tyrosine residues on substrate proteins (8). In this section, we will discuss the significance of phosphorylation modifications on several substrate proteins, including members of the mitogen-activated protein kinases family, small mothers against decapentaplegic, dynamin-related protein 1, glycogen synthase kinase-3β, and nuclear factor-kappa B, particularly concerning their roles in BPD (Figure 1A).

### Mitogen-activated protein kinases (MAPK)

MAPK, a pivotal family of proteins, includes extracellular signal-regulated kinase (ERK), c-Jun N-terminal kinase (JNK), and p38 kinase. ERK is primarily associated with cell genesis, proliferation, and differentiation, p38 is responsible for inflammation, apoptosis, and autophagy, while JNK is involved in cellular stress responses, differentiation, proliferation, and apoptosis (9). A growing body of evidence suggests that phosphorylation modifications of MAPK family members are closely intertwined with BPD. Studies have revealed that ERK phosphorylation accelerates lung fibroblast proliferation, transdifferentiation, and migration. This acceleration is achieved by expediting the cell cycle progression from the G1 to S phase and enhancing the synthesis of *α*-SMA, contributing to hyperoxia-induced lung fibrosis in neonatal rats (10, 11). Additionally, ERK phosphorylation promotes mitochondrial apoptosis and inflammatory pathways, which are significant factors in BPD (12). Conversely, decreased levels of phosphorylated ERK have been found to offer protection against hyperoxia-induced lung injury in neonatal rats (13). These findings suggest that inhibiting ERK phosphorylation may be a potential protective strategy for BPD. However, conflicting studies propose that ERK phosphorylation could mitigate hyperoxia-induced lung injury in mice by promoting angiogenesis (14). Menon et al. (15) have indicated that phosphorylated ERK levels increase early during hyperoxia exposure, potentially as a compensatory mechanism to promote angiogenesis. Nevertheless, sustained hyperoxia reduces phosphorylated ERK, hindering cell cycle progression and, consequently, pulmonary angiogenesis. Moreover, phosphorylated ERK activates cell survival signaling to mitigate hyperoxia-induced damage to lung epithelial cells (16). These contradictory findings underscore the complexity of the role played by phosphorylated ERK in BPD pathogenesis. Thus, it is essential to further investigate whether additional factors influence the impact of phosphorylated ERK in BPD, beyond the considerations related to the study population and exposure factors.

The JNK signaling pathway is closely associated with endoplasmic reticulum stress (ERS), and plays a significant role in the pathogenesis of BPD. Specifically, the phosphorylation of inositol-requiring enzyme 1 alpha (IRE1α) and JNK leads to apoptosis in lung epithelial cells associated with ERS (17). This indicates that JNK signaling activation contributes to cell death and ERS in the context of BPD. Conversely, inhibiting the phosphorylated IRE1α/JNK pathway has been shown to attenuate lung tissue inflammation, oxidative stress and apoptosis in a rat model of hyperoxia-induced lung injury (18). Phosphorylated p38 promotes this transition, which is associated with the development of lung fibrosis. This transition involves the conversion of alveolar epithelial cells (AECs) into mesenchymal-like cells, contributing to tissue scarring (19). Phosphorylated p38 inhibits the expression of claudin-18, a protein that plays a role in maintaining the integrity of the alveolar epithelial barrier. This disruption can lead to increased permeability and tissue damage (20). Activation of p38 signaling can induce ferroptosis, a type of programmed cell death characterized by lipid peroxidation and iron-dependent cell damage (21). Phosphorylated p38 can activate oxidative stress pathways and promote apoptosis in AECs, contributing to tissue injury (22). Additionally, phosphorylated p38 can enhance the expression of IL-33, a cytokine that has the ability to degrade fibronectin via its receptor neutrophil extracellular traps. This process is relevant to the pathological progression of BPD (23). Conversely, down-regulation or inhibition of phosphorylated p38 signaling has shown promise in attenuating BPD in neonatal mice by reducing inflammation (24). This suggests that targeting the p38 pathway could be a therapeutic approach to mitigate the effects of BPD.

### Small mothers against decapentaplegic (smad)

Smad proteins are crucial transcription factors that regulate gene expression. They are affected by various modifications, with phosphorylation being a key one. Phosphorylated Smad is a critical component of the transforming growth factor-β (TGF-β) pathway (25), which is closely related to BPD. Studies have identified that the TGF-β/phosphorylated Smad3 signaling pathway can trigger cell death and impair lung development, particularly in mice lacking the chaperone protein GRP78 in the endoplasmic reticulum (26). Elevated levels of phosphorylated Smad2/3 may also contribute to problems with pulmonary blood vessel development in BPD (27). Exposure to high levels of oxygen increases the phosphorylation of Smad2 and Smad3 in lung microvascular endothelial cells, leading to changes that promote endothelial-mesenchymal transition and, subsequently, pulmonary hypertension in mice (28). Therefore, targeting Smad phosphorylation shows promise as a therapeutic approach for BPD. In fact, inhibiting TGF-β-induced Smad2/3 phosphorylation has been shown to delay the development of BPD (29).

### Dynamin-related protein 1 (Drp1)

Drp1 is a key regulator of mitochondrial dynamics and undergoes various PTMs, including phosphorylation, SUMOylation, S-palmitoylation, ubiquitination, S-nitrosylation, and O-GlcNAcylation (30). Phosphorylation of Drp1, specifically at serine 616, appears to be involved in the development of BPD. Increased phosphorylation at this site is associated with mitochondrial fragmentation in lung endothelial cells and the formation of BPD-related pulmonary hypertension (BPD-PH) in rats exposed to high oxygen levels (31). Interestingly, different phosphorylation sites on Drp1 respond differently to lung injury caused by hyperoxia. Reduced phosphorylation at serine 637, for instance, is related to mitochondrial dysfunction in a mouse model of acute lung injury (ALI) (32). In contrast, in another *in vitro* experiment, hyperoxia increased phosphorylation of Drp1 at the serine 616 site to cause increased mitochondrial fragmentation in lung endothelial cells (33).

### Glycogen synthase kinase-3β (GSK-3β)

GSK-3β is a serine/threonine kinase that plays a role in various cellular processes, including cell proliferation, DNA repair, the cell cycle, and metabolic pathways, by phosphorylating target proteins (34). For instance, it can lead to sustained fibroblast activation by phosphorylating β-catenin at tyrosine 489 (6). This phosphorylated GSK-3β/β-catenin pathway can thicken alveolar septa and promote the differentiation of lung mesenchymal stromal cells (35). These are important events in the development of BPD. Rodent models further demonstrate that GSK-3β-mediated inflammation and oxidative stress play an important pathological role in hyperoxia lung injury (36). Interestingly, in a rat BPD model, increased GSK-3β phosphorylation led to lung inflammation and simplification of alveolar and pulmonary vascular structures (37). This suggests that, in addition to its role in modifying other proteins through phosphorylation, GSK-3β's own activity is influenced by phosphorylation. Specifically, increased phosphorylation at serine 9 promotes the nuclear translocation of β-catenin, which disrupts alveolar and vascular development and contributes to pulmonary hypertension associated with hyperoxia-induced lung injury in mice (38).

### Nuclear factor-kappa B (Nf-κB)

NF-κB is a transcription factor that plays a crucial role in various cellular processes, including inflammation, cell survival, and differentiation. The phosphorylation modification of NF-κB is significant in regulating these processes (39). Elevated levels of NF-κB phosphorylation were associated with inflammation and apoptosis in a rat model of BPD (40). Conversely, inhibiting NF-κB phosphorylation reduced oxidative stress and inflammation induced by hyperoxia in AECs and alleviated lung injury caused by hyperoxia in neonatal mice (41, 42). It is important to consider the roles of two upstream factors, inhibitor of κB (IκB) and inhibitor of κB kinase (IKK), in activating NF-κB. IKK becomes activated through phosphorylation and subsequently inactivates IκB by phosphorylation, leading to the nuclear translocation of NF-κB (39). In fetal lung fibroblasts, hyperoxia induces the phosphorylation of IκBα at tyrosine 42, activating NF-κB and contributing to apoptosis and oxidative stress (43). Additionally, inhibiting phosphorylated IKK/NF-κB is associated with the protective effect of IL-3 deficiency against hyperoxia-induced lung injury in mice (44).

In fact, the phosphorylation modifications of substrate proteins involved in the pathogenesis of BPD may extend beyond the examples mentioned above. For instance, in a rat model of hyperoxia-induced BPD-PH, reduced phosphorylation of endothelial nitric oxide synthase (eNOS) at the serine 1,177 site may contribute to increased pulmonary vascular tone (45). Hyperoxia also inhibits the phosphorylation of GTP-cyclohydrolase-1 at the serine 51 site, impacting tetrahydrobiopterin synthesis and potentially playing a role in hyperoxia-induced lung injury in rats (46). Clinical studies have shown that low levels of phosphorylated vasodilator-stimulated phosphoprotein, which affects cell proliferation and migration, are observed in the lung tissue of infants who succumb to BPD (47). Protein kinase B (Akt) is involved in various biological activities, such as cell survival and proliferation. Its phosphorylated form mediates forkhead box O3 (FoxO3) and E26 oncogene homologue 1 signaling, which plays an important role in counteracting hyperoxia-induced apoptosis in AECs (48, 49). However, due to space limitations, we regret that we cannot provide a comprehensive summary of all phosphorylation modifications of substrate proteins related to BPD. This remains an avenue for future research. Nevertheless, gaining a comprehensive understanding of these phosphorylation modifications of substrate proteins is likely to enhance our comprehension of the pathogenesis of BPD. Additionally, finding a balance between phosphorylation and dephosphorylation of substrate proteins may represent a potential therapeutic strategy for BPD.

### Acetylation

The process of acetylation modifications is controlled by two classes of enzymes: histone acetyltransferases, which are responsible for adding acetyl groups to lysine residues on target proteins, and histone deacetylases (HDACs), which catalyze the removal of acetyl groups (50). A growing number of studies have shown that acetylation modifications can influence the progression of BPD (Figure 1G). In a rat model of BPD, hyperoxia inhibits the activity of HDACs, leading to an increase in cytokine-induced neutrophil chemoattractant-1, which promotes inflammation and disrupts alveolar development (51). Moreover, a reduction in HDACs activity could attenuate the deacetylation of MyD88 and NF-κB, exacerbating sepsis-induced lung inflammation and impairing lung development in mice, leading to pathological changes similar to those observed in BPD (52). Conversely, enhanced HDACs activity alleviates lipopolysaccharide-induced alveolar developmental arrest in rats by inhibiting TGF-α (53). These compelling findings suggest that HDACs may serve as a promising therapeutic target for BPD. However, the role of HDACs in BPD appears to be controversial, as a previous animal study demonstrated that reduced HDACs activity could enhance the expression of acetylated histones H3 and H4, which supports recovery from hyperoxia-induced lung injury in neonatal rats (54). One possible explanation is that acetylation modifications of different target proteins may have different effects against BPD.

It is worth mentioning that the class III HDACs, which depend on NAD^+^ and are known as the sirtuins (SIRTs) family, may play an important protective role in BPD. Both human AECs and rat BPD models have shown that the activation of SIRT1 reduces hyperoxia-induced apoptosis by deacetylating p53 (55, 56). SIRT1 may also have the ability to reduce ERS associated with hyperoxia ALI in rats, but whether the exact mechanism involves the deacetylation of SIRT1 remains to be elucidated (57). Furthermore, *in vitro* and *in vivo* experiments have demonstrated that SIRTs can attenuate hyperoxia-induced AECs injury by deacetylating FoxO1 (58). This is supported by our recent study, which found that the attenuation of SIRT1's ability to deacetylate FoxO1 leads to hyperoxia-induced injury in umbilical vein endothelial cells by inhibiting autophagy (59).

### Ubiquitination

Ubiquitination is a process in which ubiquitin molecules covalently bind to target proteins, leading to their degradation in a series of steps involving E1-activating enzymes, E2-binding enzymes, and E3-ligases. Deubiquitinating enzymes (DUBs) are responsible for removing ubiquitin from ubiquitinated proteins, allowing for the dynamic reversibility of ubiquitination. Common types of DUBs include ubiquitin-specific proteases (USPs), ubiquitin C-terminal hydrolases, ovarian tumor proteases (OTUs), and others (60). Experimental studies have shown that ubiquitination modifications of specific target proteins are associated with the pathophysiology of BPD (Figure 1D). For instance, exposure to hyperoxia increases the ubiquitination modification of angiotensin-converting enzyme-2, diminishing its protective effect on lung epithelial cells (61). Additionally, nuclear factor erythroid 2-related factor 2 (NRF2) plays a protective role in BPD by regulating the antioxidant response element, a process effective only when the ubiquitinated degradation of NRF2 by kelch-like ECH-associated protein 1 (Keap1) is attenuated (62). Another target protein such as nuclear receptor subfamily 2, group F, member 2, is inhibited through ubiquitination by S-phase kinase-associated protein 2, thereby inhibiting CCAAT/enhancer-binding protein alpha (C/EBPα), which is necessary for the protection of lung cells in mice with hyperoxia-induced lung injury (63). In addition to the modification of target proteins, ubiquitination has been linked to organelle activity. Increased total ubiquitinated proteins in AECs have been shown to promote ERS-mediated apoptosis under hyperoxia exposure (64).

On the other hand, DUBs have the potential to impact BPD, even though direct evidence is lacking. Studies have revealed that USP18 and USP25 attenuate oxidative stress and inflammatory responses induced by lipopolysaccharide in human pulmonary microvascular endothelial cells and lung epithelial cells by inhibiting toll-like receptor 4/NF-κB and tumor necrosis factor receptor-associated factor 6/MAPK signaling, respectively (65, 66). Furthermore, USP22 stabilizes SIRT1 activity through deubiquitination, thereby promoting SIRT1's ability to deacetylate p53 and inhibit apoptosis (67). These findings suggest that enhancing DUB activity may contribute to the recovery of BPD. However, in BPD mouse models, He et al. (68) found that OTU3 enhances the stability of phosphatase and tensin homolog protein through deubiquitination, exacerbating lung injury and inflammatory responses. This suggests that the relationship between DUBs and BPD is complex, with the effects of DUBs on BPD depending on the action of target proteins as well as the subclasses of DUBs.

### SUMOylation

Similar to ubiquitination, SUMOylation is a process where the small ubiquitin-like modifier (SUMO) covalently attaches to lysine residues of substrate proteins in a cascade reaction catalyzed by three enzymes. In contrast, SUMO-specific proteases (SENPs) promote the maturation of the precursor SUMO and perform de-SUMOylation (69). An important consideration is that reduced SUMOylation of SIRT1 may be involved in the pathogenesis of BPD (7). There is evidence that hyperoxia upregulates SENP1 to increase the nucleoplasmic shuttling of SIRT1 and inhibits its deacetylase activity, thus promoting apoptosis in AECs (70). Conversely, the combination of budesonide and Poractant Alfa injection reduces the hyperoxia-induced increase in SENP1 in peripheral blood mononuclear cells of preterm infants, which mitigates the nucleoplasmic shuttling of SIRT1 and ultimately prevents BPD (71). These findings suggest that enhancing the SUMOylation of SIRT1 is beneficial for BPD. In addition to SIRT1, SUMOylation of other target proteins may also contribute to the pathogenesis of BPD. For example, SENP3 induces de-SUMOylation of hypoxia-inducible factor-1 alpha (HIF-1α), activating JNK and promoting M1 macrophage polarization and tissue factor-mediated coagulation cascade, leading to lipopolysaccharide-induced ALI in mice (72, 73). Moreover, fibrosis-inducing E3 ligase 1 promotes TGF-β signaling through the ubiquitinated degradation of SUMO-E3 ligase protein inhibitor of activated signal transducer and activator of transcription 4, thereby contributing to bleomycin-induced lung fibrosis in mice (74). However, it is worth noting that not all SUMOylation of target proteins is beneficial for BPD (Figure 1E). In hyperoxia-induced neonatal rat lung injury, as reported by Zhu et al. (75), the SUMOylation of C/EBPα has a negative effect on the differentiation and secretion of AECs.

### Methylation

DNA methylation, histone modifications and microRNA expression, along with the crosstalk between them, play significant roles in lung development at various stages by regulating phenotypic programming (76). As a result, epigenetic changes are pivotal in BPD development (77). Among the common complications of prematurity, BPD is one of the most likely to experience abnormal DNA methylation (78). Differential DNA methylation has been implicated in hyperoxia-exposed rat lung tissues (79), as well as during alveolar septation formation in mice and humans (80). Notably, in rats with hyperoxia-induced lung injury, DNA methyltransferase (DNMT) 3b-mediated DNA methylation and enhancer of zeste homolog 2-mediated tri-methylation of lysine 27 on histone H3 decrease runt-related transcription factor 3 proteins associated with lung epithelial and vascular development, possibly contributing to the pathogenesis of BPD (81). Furthermore, hyperoxia enhances DNA hypermethylation of *TGF-*β pathway-related genes and immune system-related genes, particularly the *PI3K-AKT* pathway, affecting alveolar development and later abnormal responses to respiratory infections in mice (82, 83). Additionally, DNMT-mediated methylation of certain microRNA promoters is associated with BPD severity (84). These findings suggest that targeting epigenetics may be a potential strategy to alleviate BPD (Figure 1F). Indeed, it has been shown that vitamin D promotes the methylation status of vitamin D-responsive elements in the interferon-*γ*-promoter region, thereby inhibiting interferon-γ expression and attenuating lipopolysaccharide-induced BPD in rats (85). In BPD mouse models, the application of DNA methylase inhibitors potentially reduces phosphorylated Smad2/3 and increases surface-active protein C levels, thereby improving alveolarization (86). Furthermore, DNA methylation inhibitors reverse hyperoxia-induced aberrant methylation of the cell cycle inhibitor *p16* gene, thereby attenuating pulmonary fibrosis in neonatal rats (87).

### Glycosylation

Glycosylation refers to the process of attaching glycans to proteins and lipids, which is carried out by glycosyltransferases. Conversely, glycosidases are responsible for breaking down glycosidic bonds, leading to de-glycosylation (88). Glycosylation plays a crucial role in various biological processes, including the secretion and translocation of proteins. It also ensures the proper folding of proteins and shields them from degradation by proteases (89). There is evidence indicating that exposure to high levels of oxygen, known as hyperoxia, can temporarily disrupt the glycosylation balance in mouse lung microvascular endothelial cells. This disruption may impact cellular interactions and signal transduction (90). Furthermore, a protein called endoplasmic reticulum protein 57, associated with glycosylation modifications, has been found to contribute to hyperoxia-induced apoptosis in human endothelial cells by inhibiting GRP78 and activating caspase-3 (91). Clinical studies have shown that preterm infants, especially those born at less than 28 weeks of gestational age, exhibit increased glycosylation of IgG Fc in their peripheral blood. This increased glycosylation may play a role in the development of inflammatory diseases such as BPD (92).

O-GlcNAcylation is a specific type of glycosylation involving the binding of O-linked β-N-acetylglucosamine (O-GlcNAc) to serine or threonine residues of substrate proteins. O-GlcNAc transferase and O-GlcNAcase are enzymes responsible for adding and removing O-GlcNAcylation, respectively (93). This process is implicated in various essential cellular activities, including gene expression, signal transduction, cell cycle regulation, nutrient sensing, protein homeostasis, cellular stress response and neuronal function (94). O-GlcNAcylation is particularly closely related to oxidative stress (95). For instance, reduced O-GlcNAcylation of lung tissue proteins has been suggested as an essential response to injury in sepsis-induced ALI models in mice (96). Conversely, O-GlcNAcylation of specific target proteins, like SIRT1 at the serine 549 site, can enhance its deacetylation activity (97), promoting recovery in BPD by reducing apoptosis (56). However, in cases of hyperoxia-induced injury in AECs, increased O-GlcNAcylation of the mitophagy regulator Parkin can disrupt mitochondrial homeostasis and lead to apoptosis (98) (Figure 1B). This indicates that O-GlcNAcylation can have both positive and negative effects in BPD, depending on the specific substrate proteins, and requires further investigation to understand its precise mechanisms of action.

### Glycation

In contrast to glycosylation, glycation (also known as the Maillard Reaction) is a non-enzymatic reaction where free sugars covalently bind to proteins. This reaction produces advanced glycation end products (AGEs), which are important stabilizing substances (99). The receptor for AGEs (RAGE) is abundant in AECs, where it plays a positive role in lung development. However, under pathological conditions, RAGE interacts with its ligands, including AGEs, leading to inflammation and oxidative stress that can induce lung diseases (100). RAGE makes the developing lung more susceptible to the harmful effects of hyperoxia (101). Studies involving RAGE-deficient mice have shown delayed hyperoxia-induced mortality and ALI (102). RAGE also disrupts the alveolar-capillary barrier (103) and promotes inflammatory signals, such as high mobility group box 1 (104) and NF-κB (105), which are significant mechanisms for RAGE's involvement in BPD. Clinical studies have identified serum RAGE as a promising biomarker for predicting BPD, suggesting that targeting RAGE may be a new approach to treating lung diseases (106).

### S-glutathionylation

S-Glutathionylation is a process where mixed disulfides are formed between glutathione and specific cysteine residues on target proteins (5). It plays a crucial role in regulating cellular redox signaling. For instance, in lipopolysaccharide-induced ALI in mice, S-glutathionylation of macrophage fatty acid-binding protein 5 at cysteine 127 is enhanced. This modification activates peroxisome proliferator-activated receptor (PPAR), facilitating the cells to resist oxidative stress (107). Significant changes in S-glutathionylation of proteins are observed in mouse models of hyperoxia-induced lung injury, underlining its critical role in BPD (108). S-glutathionylation of the substrate protein Keap1 is necessary for activating the antioxidant NRF2, which may protect experimental neonatal rats against BPD (109) (Figure 1C). However, S-glutathionylation of proteins can also have adverse effects under certain conditions. For example, S-glutathionylation of certain apoptotic proteins in lung epithelial cells has potential pathophysiological significance in pulmonary fibrosis (110). Additionally, hyperoxia-induced S-glutathionylation of manganese superoxide dismutase increases mitochondrial oxidative stress in macrophages, leading to increased mortality in mice with ventilator-associated pneumonia (111). These findings suggest that the process by which S-glutathionylation of proteins affects BPD may involve a complex regulatory network, and more evidence is needed to elucidate the specific mechanisms.

Glutaredoxin (Grx), primarily responsible for deglutathionylation, also plays a role in cellular redox signaling. *Grx1* gene deficiency has been found to stabilize HIF-1α and inhibit NF-κB, promoting angiogenesis and reducing apoptosis to alleviate hyperoxia-induced lung injury in mice (112). However, another study suggests that Grx deficiency may increase the expression of TGF-β in respiratory epithelial cells, potentially raising the risk of developing fibrotic lung disease (113). The differing effects of Grx may depend on oxidative inducers and the specific substrate proteins involved.

### S-nitrosylation

S-nitrosylation is a reaction where the nitrosyl group of nitric oxide (NO) covalently binds to the thiol group of cysteine, forming S-nitrosothiol. This process promotes pulmonary vasodilation but can lead to alveolitis and constriction of pulmonary artery smooth muscle in the presence of high inhaled NO doses and oxidative stress. Therefore, maintaining a dynamic balance of S-nitrosylation is beneficial for alveolar integrity and NO-mediated vasodilation (114). S-nitrosoglutathione (GSNO) is a critical donor of NO that promotes S-nitrosylation, while S-nitrosoglutathione reductase (GSNOR) is responsible for de-S-nitrosylation by acting on GSNO (5). Previous studies have shown that GSNO contributes to airway relaxation in BPD mice, whereas GSNOR is associated with increased airway hyperresponsiveness related to BPD (115, 116). Knockout of the alcohol dehydrogenase-5 gene encoding GSNOR has been shown to ameliorate BPD and BPD-PH (117). Clinical studies have revealed high expression of GSNOR in the airways and pulmonary vasculature of BPD patients (117), suggesting its significance in BPD pathogenesis and its potential as a therapeutic target for BPD-PH.

Thioredoxin (Trx) is another important regulator of S-nitrosylation, and exposure to hyperoxia can impact Trx function. The inhibition of Trx can interfere with heat shock protein 90-mediated oxidative responses, negatively affecting cell survival signaling pathways associated with BPD (118). Conversely, promoting Trx expression can prevent hyperoxia-induced loss of uncoupling protein 2 by activating the mitogen-activated protein kinase kinase 4/p38/PPAR-gamma coactivator 1 alpha pathway, thus reducing superoxide anion production (119). Trx also suppresses hyperoxia-induced inflammatory responses (120). These effects of Trx have been shown to be beneficial in mouse models of BPD. Furthermore, Trx enhances the ability of mesenchymal stromal cells (MSCs) to resist hyperoxia injury by inhibiting apoptosis-regulating kinase-1/p38-mediated apoptosis signaling. This improvement in MSC efficacy holds promise for treating BPD (121).

## Conclusion

BPD is indeed a significant and challenging complication in preterm infants, and gaining a deeper understanding of the molecular mechanisms underlying its pathogenesis is crucial. PTMs influence almost all cellular biological processes by altering the function of substrate proteins in response to environmental changes. Investigating the relationship between PTMs and BPD is a promising avenue of research, as it can lead to early detection of BPD and the development of innovative therapeutic strategies. In this article, we have discussed the significance of several common PTMs, including phosphorylation, acetylation, ubiquitination, SUMOylation, methylation, glycosylation, glycation, S-glutathionylation, and S-nitrosylation, in the context of BPD (Figure 1). However, several important questions remain unanswered. Firstly, many substrate proteins can undergo multiple PTMs, and understanding how crosstalk between different PTMs influences BPD requires further investigation. Secondly, the same PTMs can act on different substrate proteins, resulting in diverse effects on BPD. Deciphering the roles of both PTMs and substrate proteins in influencing BPD is a complex task. Finally, establishing a comprehensive network of PTMs related to BPD and harnessing PTMs to develop novel diagnostic markers and therapeutic targets for BPD is an intriguing prospect that holds the potential to transform our approach to managing this condition.
